# Antifungal Efficacy of Plasma-Activated Liquids against *Candida albicans*: A Potential Alternative for Oral Candidiasis Treatment

**DOI:** 10.1021/acsomega.5c06368

**Published:** 2025-10-29

**Authors:** Sabrina de Moura Rovetta-Nogueira, Felipe de Souza Miranda, Diego Morais da Silva, Michaela Shiotani Marcondes, Rodrigo Sávio Pessoa, Cristiane Yumi Koga-Ito

**Affiliations:** † Institute of Science and Technology, 28108 São Paulo State University (UNESP), Avenida Engenheiro Francisco José Longo, 777 São José dos Campos, Brazil; ‡ Plasmas and Processes Laboratory, Department of Physics, 74360 Aeronautics Institute of Technology, Praça Marechal Eduardo Gomes 50, São José dos Campos 12228-900, Brazil

## Abstract

The increasing incidence of oral candidiasis refractory to conventional antifungal treatments, coupled with limited therapeutic alternatives, underscores an urgent need for novel interventions. Plasma-activated liquids (PALs), generated via nonthermal plasma, have shown antimicrobial promise, yet their antifungal efficacy, particularly against *Candida albicans*, remains understudied, and no investigations have addressed their use in oral candidiasis. In this study, PALs were produced using a gliding-arc plasma reactor with argon and compressed air (pure and mixed) at gas flows of 1.5 and 3.0 L/min, and an average power range of 25–60 W. Two liquid substrates, distilled water and 0.9% sodium chloride (saline) solution, were plasma-activated and characterized in terms of physicochemical parameters (pH, ORP, TDS, conductivity) and reactive oxygen and nitrogen species (RONS), including H_2_O_2_, NO_2_^–^, NO_3_^–^, HNO_2_, and O_3_, via spectrophotometry and reactive-strip analysis. The antifungal activity of PALs was assessed against *C. albicans* in both planktonic and biofilm forms. After 30 min exposure, argon-activated saline (S1) and distilled water (D1), and argon–air activated distilled water (D2), achieved ∼50% reduction in planktonic viability; only S1 and D1 maintained significant effects on 24- and 48 h biofilms. Moreover, PALs frozen at −18 °C for 24 h retained antifungal efficacy. Cytotoxicity assays using Vero cells confirmed that D1 and S1 were nontoxic over both immediate and 24 h exposures (viability >70%). In sum, argon-derived PALs based on distilled water and saline exhibited selective antifungal and antibiofilm activity against *C. albicans*, without toxicity to mammal cells, highlighting their potential as adjunctive therapies in candidiasis management. These findings support further optimization for clinical applications.

## Graphical abstract

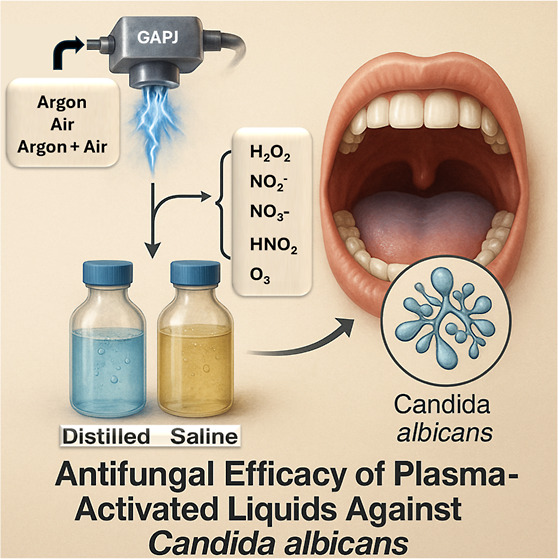

## 1 Introduction

Plasma technology has been extensively studied and applied in various fields, including the sterilization of medical equipment, the food industry, surface treatment, grain sterilization, internal sterilization of sealed packaging, as well as in textile and automotive industries, and water treatment.^1–10^

Various disciplines, including energy, photonics, telecommunications, space exploration, and materials science, have explored plasma-based technologies. More recently, plasma has gained increasing attention in the biomedical field, where interdisciplinary efforts involving engineering, physics, chemistry, biology, dentistry, and medicine have produced compelling in vitro and in vivo evidence of its efficacy. Notably, plasma has shown promise in the noninvasive and painless treatment of persistent infections and several types of cancer.^4,11–22^

Plasma is defined as a partially ionized gas composed of electrons, ions, excited species, free radicals, and photons, generated by electromagnetic fields produced by microwave sources, radio frequency generators, or direct/alternating current applied to noble gases (e.g., argon, helium) or molecular gases (e.g., oxygen, nitrogen).^8,23–25^ Laroussi^26^ was the first to report the bactericidal effect of cold plasma, which has since led to a large body of research investigating its antimicrobial potential,^27–34^ and the physicochemical mechanisms underlying its therapeutic effects.^8,23,25,35–41^

In recent years, indirect applications of plasma, particularly the exposure of liquids to plasma discharges, have shown encouraging results.^2,42,43^ The antimicrobial activity of plasma-activated water (PAW) against *Escherichia coli* has been associated with the generation of oxidative environments.^44^ In general, plasma–liquid interactions lead to the formation of hydrogen peroxide, nitrites, nitrates, and other oxidizing agents, which can stimulate the generation of reactive oxygen and nitrogen species (RONS) in biological systems.^43,45–48^ This approach has recently been evaluated for medical applications, offering numerous advantages: plasma is not applied directly to biological tissue, but rather through preactivated liquids, making the method safer, more flexible, and adaptable to diverse clinical contexts. Bhatt et al.^49^ reported the inhibitory effects of plasma-activated solutions on biofilms formed by MRSA, *Staphylococcus epidermidis*, *Pseudomonas aeruginosa*, and *Candida albicans*, suggesting potential use in lock therapy for catheter-associated infections.

Despite these advances, the antifungal effects of plasma-based treatments, particularly in liquid form, remain less explored than their antibacterial counterparts. Fungal infections have emerged as a significant global health concern, especially among immunocompromised individuals. The incidence of fungal infections has risen considerably in recent decades,^50^ with *Candida* species being especially relevant in clinical settings.^51^ Oral candidiasis is one of the most common mucosal fungal infections, frequently affecting patients undergoing chemotherapy, radiotherapy, organ transplantation, or living with HIV/AIDS. These infections may be superficial or systemic; in the latter, fungal dissemination can lead to candidemia with mortality rates ranging from 30% to 50%.^52,53^ Importantly, the oral cavity can serve as a reservoir for systemic dissemination, particularly in vulnerable patient populations.^54–56^

Current antifungal therapies, including azoles and polyenes, are often limited by cytotoxic effects, limited activity against biofilms, drug–drug interactions, and the emergence of resistant fungal strains. Furthermore, increasing cases of therapeutic resistance to conventional antifungal agents have been documented.^57,58^ This growing resistance crisis, combined with the limited spectrum of activity and potential side effects of existing drugs, underscores the urgent need for safer and more effective antifungal strategies.^59–64^ Plasma-based technologies offer a promising route for the treatment of fungal infections, especially considering the limitations of existing therapies.^59,65^ An additional concern is the enhanced resistance of fungal biofilms relative to planktonic cells. Biofilms can be up to 1000 times more resistant, making treatment particularly challenging.^64,66,67^

The inhibitory effects of cold atmospheric plasma jets on *C. albicans* have been documented in several studies, demonstrating reductions in biofilm adhesion, filamentation, and viability.^35,41,68–74^ In vivo application in murine models has shown favorable antifungal and anti-inflammatory outcomes, with low tissue toxicity.^75^ According to Liu et al.^76^ and Ma et al.,^77^ one of the main advantages of plasma-activated liquids (PALs) over direct plasma exposure is improved safety, as PALs avoid electric fields, charged particles, and thermal effects while retaining therapeutic activity through long-lived reactive species.^76,78,79^

Nevertheless, studies specifically addressing the antifungal potential of PALs against *C. albicans* remain scarce. While some reports indicate limited success in reducing fungal viability,^20,78,80,81^ the variability in plasma sources, activation parameters, and liquid composition complicates reproducibility and standardization.

In this context, the present study aims to investigate the antifungal effects of plasma-activated liquids (PALs) generated by a gliding arc plasma reactor, with a particular focus on *C. albicans*, the primary etiological agent of oral candidiasis. Unlike prior reports, we systematically evaluate PALs produced from distilled water and saline solution activated by argon, compressed air, and their mixtures, and assess their effects on both planktonic and biofilm forms of *C. albicans*. The PALs are also evaluated for physicochemical properties, reactive species content, and toxicity to mammal cells. Our results reveal that specific PALs exhibit significant antifungal activity against *C. albicans*, particularly in biofilm states, while maintaining biocompatibility. These findings offer new insights into the use of PALs as adjunctive therapies in the management of candidiasis, especially in cases of drug resistance.

## 2 Materials and Methods

### 2.1 Gliding Arc Plasma Jet Reactor

The experimental setup for the gliding arc plasma jet (GAPJ) consisted of a plasma reactor, a high-voltage power supply, an oscilloscope, a gas source, and a substrate support, as illustrated in Figure 1. Plasma was generated inside a forward vortex flow reactor (FVFR). The distance between the reactor and the liquid surface was maintained at 0.5 cm. In each experiment, 40 mL of liquid was treated.

**Figure 1:**
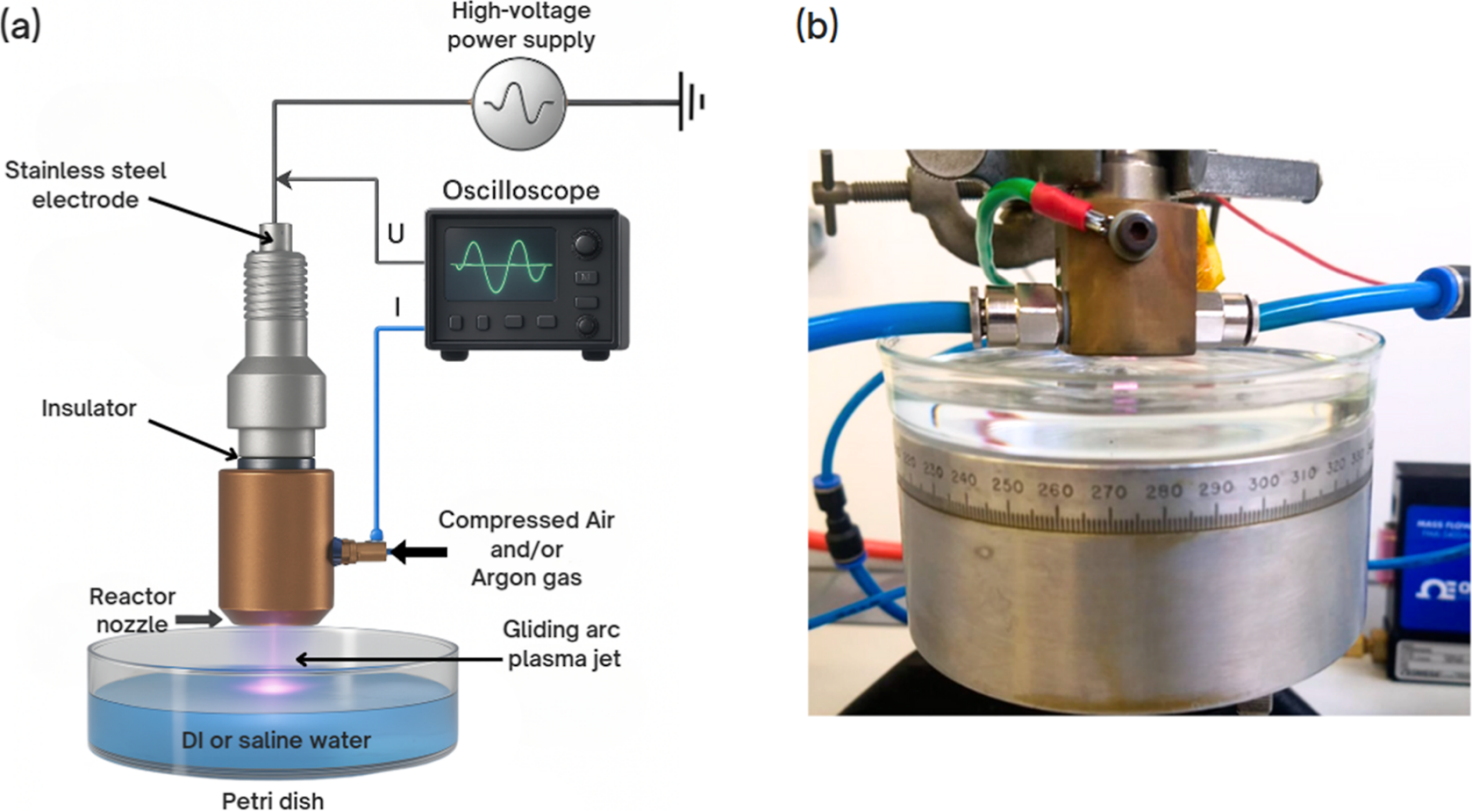
Experimental setup for the generation of plasma-activated liquids (PALs) using a gliding arc plasma jet (GAPJ). (a) Schematic showing the plasma reactor, high-voltage power supply, oscilloscope, and gas inputs (argon and/or compressed air). Plasma is applied to 40 mL of liquid in a Petri dish, with a fixed distance of 0.5 cm between the nozzle and the liquid surface. (b) Photograph of the reactor positioned above the Petri dish during operation.

Two gas sources were used: argon (99.5%) and compressed air, the latter supplied by a Schulz CSD 9/50 air compressor (Joinville, SC, Brazil). Initially, both gases were used at a flow rate of 1.5 L/min, individually and in mixtures (1.5 L/min each). Based on the microbiological outcomes, additional tests were performed at an increased flow rate of 3.0 L/min for each gas and for their mixtures, which was then adopted as the standard flow rate for the experimental phase.

Electrical power was supplied by a high-voltage source (model Arternis 0215, Inergiae, Florianópolis, SC, Brazil), operating at 20 kHz. Discharge voltage and current were measured using a high-voltage probe (Tektronix P6015A, Tektronix, Beaverton, OR, USA) and a current probe (Agilent N2869B, Agilent, Santa Clara, CA, USA), respectively. Signals were recorded on a digital oscilloscope (Keysight DSOX1202A, Keysight, Santa Rosa, CA, USA). The average discharge power was 25.4 W for argon, 60.6 W for compressed air, and 33.4 W for the argon-air mixture.

### 2.2 Quantification of Reactive Species in Plasma-Activated Liquids

#### 2.2.1 Quantification by Spectrophotometry

Reactive oxygen and nitrogen species (RONS) in PALs were detected using a UV–vis spectrophotometer (Evolution 201, Thermo Scientific, Waltham, MA, USA). Absorbance was measured across the 190–900 nm range with a 0.2 nm spectral resolution and a scanning speed of 120 nm/min. Samples were placed in 3.5 mL quartz cuvettes (K22-135Q, Kasvi, São José dos Pinhais, SP, Brazil) with a 10 mm optical path.

A blank spectrum was first recorded using an empty cuvette, followed by one containing deionized water to ensure baseline correction. Clean cuvettes were then filled with PAL aliquots to acquire relative UV absorption spectra, following Dascalu et al.^82^ This calibration was repeated for PALs generated from 0.9% NaCl saline solution.

#### 2.2.2 Quantification by Reactive Strips

Quantification of specific RONS was performed using Quantofix reactive strip kits for hydrogen peroxide, nitrite, and nitrate. Strips were immersed in the samples for 15 s and analyzed per manufacturer instructions. Concentration ranges were: 0.5–25 mg/L (H_2_O_2_), 1–80 mg/L (NO_2_^–^), and 10–500 mg/L (NO_3_^–^). Colorimetric comparison with reference charts provided concentration estimates.

Ozone levels were measured using an Exact Micro 20 multiparameter photometer (Industrial Test System, USA), with readings taken after 5 min.

### 2.3 Physicochemical Analysis of Plasma-Activated Liquids and Controls

Parameters including pH, oxidation–reduction potential (ORP), electrical conductivity, and total dissolved solids (TDS) were measured before and after plasma activation using a Metrohm 913 benchtop meter.

### 2.4 PALs: Transport, Storage, and Stability

Plasma-activated liquids prepared with distilled water and 0.9% saline were stored at approximately ±18 °C in 3 mL cryogenic tubes. Prior to use, samples were thawed for 15 min at 16–19 °C under sterile conditions in a laminar flow cabinet.

PALs were categorized according to the liquid substrate and the type of activating gas, as summarized in Table 1. A total of 20 experimental groups were evaluated: four groups at a flow rate of 1.5 L/min (including controls) and 16 at 3.0 L/min.

**Table 1:** Experimental Groups Based on Liquid Substrate and Activating Gas

distilled water	Saline
CD: Control	CS: Control
D1: Argon	S1: Argon
D2: Ar + Air	S2: Ar + Air
D3: Air	S3: Air

### 2.5 Strains and Growth Conditions

The reference strain *C. albicans* ATCC 18804 was stored in Sabouraud broth with 20% glycerol at −80 °C. Inocula were prepared from cultures grown on Sabouraud dextrose (SD) agar at 37 °C for 24 h under aerobic conditions. Suspensions were prepared in sterile 0.9% NaCl and standardized spectrophotometrically at 550 nm: OD = 0.380 (10^6^ cells/mL) for planktonic and OD = 0.830 (10^7^ cells/mL) for biofilm assays.

### 2.6 Effect of LAPs on Planktonic Cells of *C. albicans*

Aliquots of 1 mL of suspensions containing 10^7^ cells were centrifuged at 1500 rpm for 15 min. After supernatant removal, pellets were resuspended in 1 mL of PAL. Suspensions were vortexed and exposed to PALs for 10 or 30 min. Serial dilutions (10^–1^ to 10^–6^) were prepared, and dilutions 10^–3^ to 10^–5^ were plated on SD agar using the Miles and Misra method.^83^ Colony forming units (CFUs) were counted after 24 h.

### 2.7 Effect of PALs on *C. albicans* Biofilms

#### 2.7.1 24 h Biofilms

Biofilms were formed in 96-well plates with 250 μL of inoculum per well, incubated at 37 °C, 75 rpm for 90 min for adhesion. Wells were washed with physiologic solution (NaCl 0.9%), and 250 μL of RPMI was added. After 24 h, supernatants were removed, the wells were washed, and PALs (D1, S1) or controls were added for 30 min. Biofilms were scraped, resuspended, serially diluted, and plated on SD agar for CFU quantification.

#### 2.7.2 48 h Biofilms

Tests followed the same protocol as for 24 h biofilms, using the S1 group.

### 2.8 Cytotoxicity Test of Plasma-Activated Liquids

Cytotoxicity was assessed according to ISO 10993–5:2009 using Vero cells cultured in DMEM with 1% penicillin/streptomycin and 10% FBS at 37 °C in 5% CO_2_. Cells were seeded at 8 × 10^3^ cells/well and incubated for 24 h. Test solutions (PALs and controls) were applied at a 1:1 ratio with medium (total volume 200 μL/well) for 30 min. Cells were then washed and incubated with fresh medium for 24 h.

The MTT assay was used to assess viability. After 24 h, 100 μL of MTT was added, incubated for 2 h, followed by DMSO addition to dissolve formazan. Absorbance was read at 570 nm. Viability below 70% was considered cytotoxic.

### 2.9 Data Analysis

Data were analyzed using Origin Lab 8.5. Normality was tested with the Shapiro–Wilk test. If normality was confirmed, one-way ANOVA was applied. When significant, Tukey’s post hoc test identified differences. Significance was set at *p* < 0.05. Statistical analysis was applied to all biological assays, including CFU counts from planktonic and biofilm tests and cytotoxicity data. Physicochemical and spectrophotometric measurements were evaluated using descriptive statistics (mean and standard deviation), and replicate variation is indicated in figures or tables. These data sets were used to identify trends and correlations rather than test statistical hypotheses.

## 3 Results

### 3.1 Physicochemical Evaluation of Plasma-Activated Liquids

The evaluation of the physicochemical parameters revealed that pH decreased in all experimental groups following plasma activation (Table 2).

**Table 2:** Physicochemical Parameters of Liquids Before and After Plasma Activation Using Different Gases and Flow Rates^a^

groups		pH	TDS (mg/L)	ORP (mV)	σ (μS/cm)
Flow 1.5 L/min	DC	6.86	25.95	37.2	52.0
	D1	6.19	43.90	76.8	125.9
	D2	6.20	44.56	125.8	155.8
	D3	5.77	57.89	157.2	215.1
Flow 3.0 L/min	SC	4.73	42,553	123.1	107,200
	S1	4.24	44,401	153.7	111,100
	S2	2.56	52,827	251.9	130,100
	S3	2.47	53,027	257.2	126,700
Flow 3.0 L/min	DC	5.80	26.76	51.0	58.0
	D1	4.05	137.50	149.4	275.5
	D2	2.96	166.80	229.7	405.5
	D3	2.82	159.90	237.7	384.5

a Legend: TDS: Total dissolved solids; ORP: Oxidation–reduction potential; σ: electrical conductivity; pH: hydrogen potential. DC: nonactivated distilled water control; D1: distilled water activated with argon plasma; D2: distilled water activated with argon and air; D3: distilled water activated with air plasma; SC: nonactivated saline control; S1: Saline activated with argon plasma; S2: Saline activated with argon and air; S3: Saline activated with air plasma.

The most pronounced pH reduction occurred in the groups treated with compressed air plasma (S3 and D3). In contrast, argon-only activated groups exhibited a more moderate pH decrease. The groups activated with a combination of argon and compressed air (D2 and S2) also showed notable pH reductions, likely due to the influence of the air component. Each measurement of pH, TDS, ORP, and conductivity was performed in triplicate, and the values reported in Table 2 represent the mean of three independent measurements. As these physicochemical parameters were used to identify general trends rather than to test specific hypotheses, descriptive analysis was applied without inferential statistical testing.

In addition, TDS, electrical conductivity, and ORP increased in all groups after plasma exposure. These parameters demonstrated an inverse relationship with pH: as the pH decreased, TDS, conductivity, and ORP values increased for both distilled water and 0.9% saline PALs, as illustrated in Figure 2.

**Figure 2:**
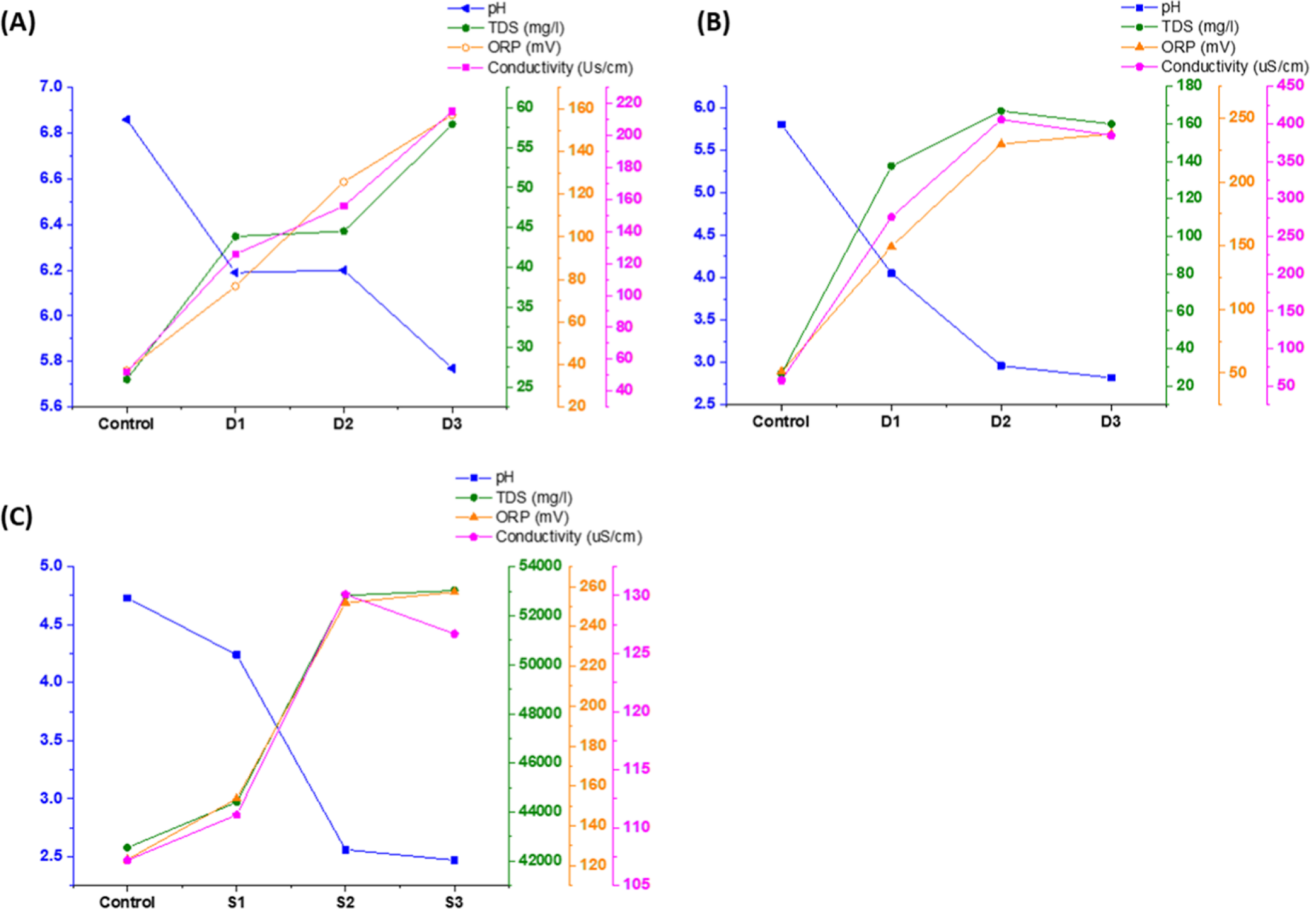
Physicochemical parameters of plasma-activated liquids (PALs). Graphs show the variation in pH, total dissolved solids (TDS), oxidation–reduction potential (ORP), and electrical conductivity of (a) distilled water activated at 1.5 L/min (D1, D2, D3), (b) distilled water activated at 3.0 L/min (D1, D2, D3), and (c) 0.9% saline solution activated at 3.0 L/min (S1, S2, S3). Controls refer to nonactivated liquids. All PALs showed a decrease in pH and an increase in TDS, ORP, and conductivity after activation, with the most pronounced changes observed in compressed air-treated samples (D3 and S3).

### 3.2 UV–Vis Spectroscopy Analysis

In Figure 3, the deconvoluted UV absorbance spectra of PALs reveal distinct patterns of RONS formation depending on the gas composition used during plasma activation.

**Figure 3:**
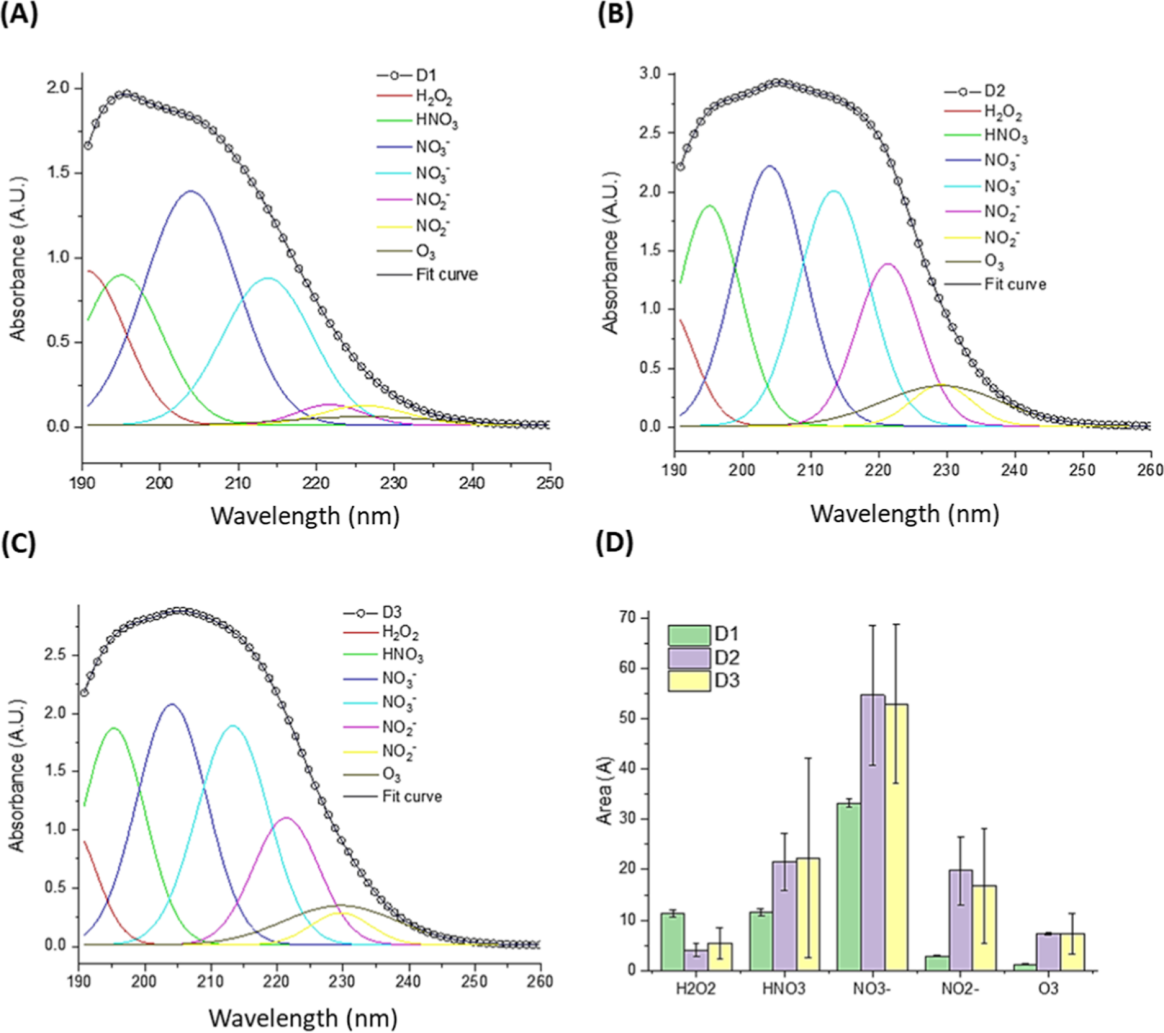
Deconvoluted UV absorbance spectra of plasma-activated liquids (PALs) based on distilled water, highlighting the formation of reactive oxygen and nitrogen species (RONS) after exposure to different plasma gas compositions: (a) D1—argon plasma; (b) D2—argon and compressed air; (c) D3—compressed air plasma. Individual colored curves correspond to specific RONS: hydrogen peroxide (H_2_O_2_), nitric acid (HNO_3_), nitrate (NO_3_^–^), nitrite (NO_2_^–^), and ozone (O_3_). The black line represents the fitted composite spectrum. (d) Quantitative comparison of RONS content, shown as areas under the deconvoluted spectral peaks, with error bars indicating standard deviations from replicate analyses.

Figure 3a–c show that although the general spectral profiles share a broad absorbance band between 190–250 nm, the underlying contributions from individual species differ considerably among the samples D1 (argon), D2 (argon + compressed air), and D3 (compressed air). This observation confirms that gas composition is a critical factor governing the plasma-induced chemistry in liquids.

The fitted curves indicate the presence of key long-lived RONS, including hydrogen peroxide (H_2_O_2_), nitrous acid (HNO_2_), nitrite (NO_2_^–^), nitrate (NO_3_^–^), and ozone (O_3_), consistent across all conditions. Notably, the intensity and area of the peaks vary significantly. Sample D1 (argon-only plasma) exhibits lower intensities for nitrogen-based species, as expected due to the absence of nitrogen in the feed gas. In contrast, D2 and D3, both containing compressed air, exhibit enhanced formation of NO_3_^–^ and NO_2_^–^, with D3 (compressed air) showing the highest overall concentration of nitrogenous RONS, particularly nitrate. This reflects the increased availability of nitrogen species and the elevated energy density in air plasmas that favor multistep oxidation reactions.

Figure 3d provides a quantitative comparison of the areas under the deconvoluted peaks, reinforcing the qualitative differences observed in the spectra. D3 plasma, generated using only compressed air, yields the largest amounts of NO_3_^–^ and NO_2_^–^, indicating a stronger oxidative environment likely due to enhanced NOx formation in the gas phase followed by dissolution into the liquid phase. O_3_ and H_2_O_2_ also appears, with higher levels of O_3_ observed in D2 compared to D3, suggesting a potential competitive formation mechanism between nitrogen oxides and ozone under air-rich plasma conditions. All UV–vis measurements and deconvolution analyses were performed using independently prepared PAL samples (*n* = 3). Results shown in Figure 3d represent average areas under the fitted curves, with error bars indicating standard deviations. These values were used to compare relative RONS abundance across groups, but no inferential statistical analysis was applied, as the objective was qualitative and semiquantitative spectral interpretation.

For the group corresponding to the saline solution, the deconvolution of UV absorbance spectra (Figure 4) reveals the presence and relative contributions of long-lived RONS formed during plasma activation of 0.9% NaCl solutions.

**Figure 4:**
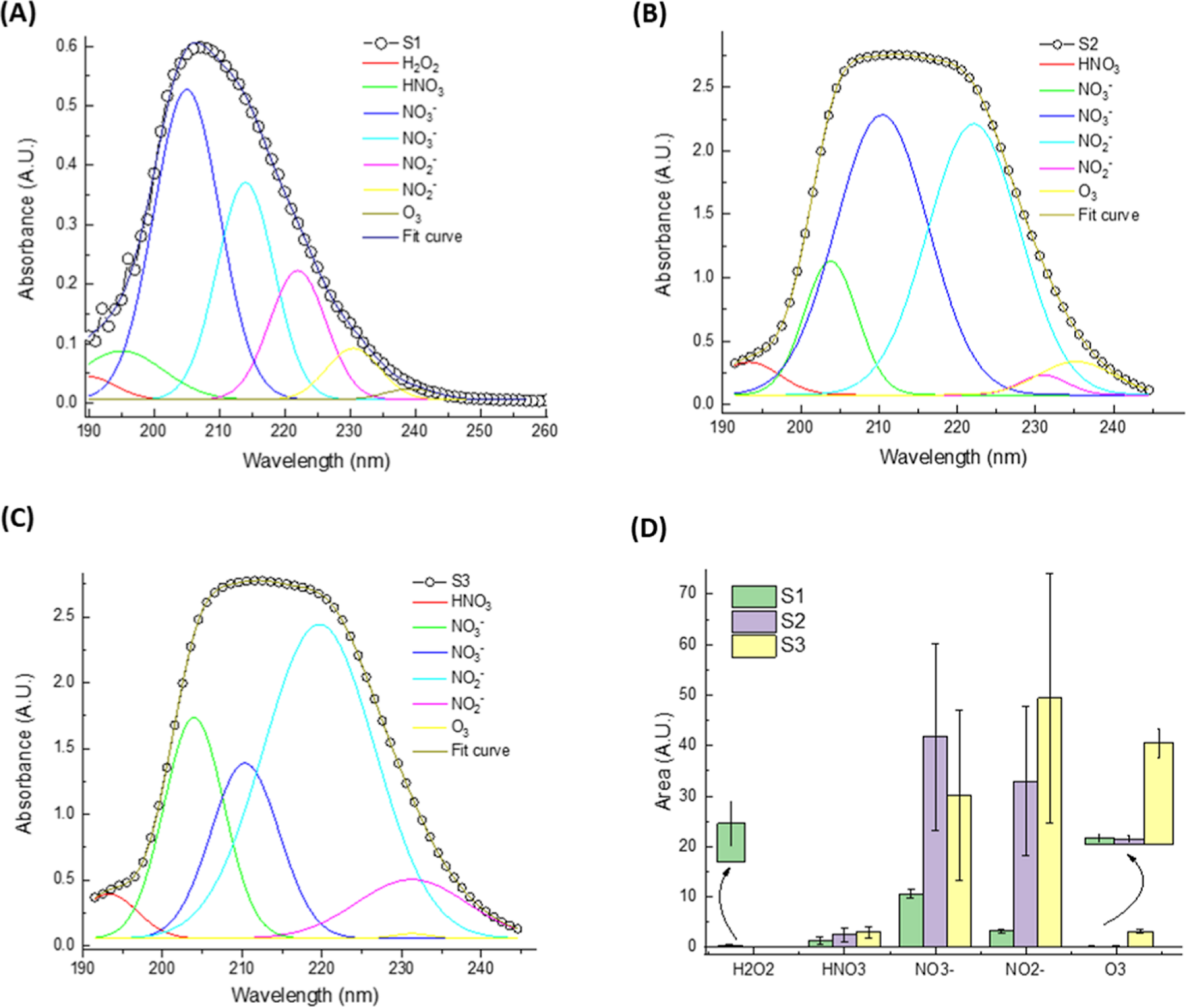
UV absorbance deconvolution of plasma-activated liquids (PALs) based on 0.9% saline solution, highlighting the reactive oxygen and nitrogen species (RONS) formed following plasma treatment: (a) S1—activated with argon plasma; (b) S2—activated with argon and compressed air; (c) S3—activated with compressed air plasma. Individual colored curves represent the spectral contribution of hydrogen peroxide (H_2_O_2_), nitric acid (HNO_3_), nitrate (NO_3_^–^), nitrite (NO_2_^–^), and ozone (O_3_); the black line represents the fitted spectrum. (d) Areas under the curve for each species, indicating their relative abundance and variability across the experimental groups.

Figure 4a–c show that even in the presence of ionic content, the spectral features remain distinct and reproducible across the different gas compositions used: S1—argon plasma; S2—argon + compressed air; and S3—compressed air plasma.

Similar to the behavior observed for distilled water, the overall spectral shape appears as a broad band centered between 190 and 240 nm, but the underlying deconvoluted peaks indicate considerable differences in species composition and intensity. Notably, Figure 4a, corresponding to S1 (argon plasma), displays the lowest overall RONS absorbance, particularly for nitrogen-containing species, which is consistent with the inert nature of argon and the absence of nitrogen in the discharge. Nevertheless, small quantities of H_2_O_2_, NO_2_^–^, and O_3_ are still observed, possibly from background air diffusion and secondary reactions.

In Figure 4b, representing S2, the use of argon and compressed air leads to a marked increase in both NO_3_^–^ and NO_2_^–^ peaks, suggesting enhanced nitrogen species oxidation via air admixture. However, the most significant RONS production is evident in Figure 4c (S3), where compressed air is used. This sample shows a strong nitrate peak and substantial contributions from ozone and nitrite, reinforcing the pattern seen in distilled water but with slightly different proportions. The high ionic strength of the saline matrix may influence the solubility and stabilization of certain species, especially NO_2_^–^ and O_3_.^84,85^ All absorbance spectra were acquired from independently generated samples (*n* = 3), and the deconvoluted spectral areas shown in Figure 4d reflect average values with standard deviations. Given the analytical nature of this characterization, these results were interpreted descriptively without applying inferential statistical tests.

Figure 4d quantitatively illustrates the integrated area under the fitted peaks for each species across the samples. NO_3_^–^ dominates in all cases, particularly in S2 and S3, which correlates with the nitrogen availability in the plasma gas. Interestingly, H_2_O_2_ is present in lower quantities in saline compared to distilled water. The O_3_ levels in S3 remain significant, indicating that the saline matrix does not inhibit its formation.

### 3.3 Quantification of Reactive Species via Test Strips

The concentrations of reactive species detected in the PALs by commercial reactive test strips are summarized in Table 3. Hydrogen peroxide concentrations were higher in PALs generated using only argon plasma (D1 and S1), ranging from 2.0 to 5.0 mg/L. In contrast, groups treated with compressed air or mixed gas plasmas (D2, D3, S2, S3) exhibited lower H_2_O_2_ levels (∼0.5 mg/L), but significantly higher concentrations of nitrite (250–500 mg/L) and nitrate (40–80 mg/L). These results suggest that the use of compressed air enhances the formation of reactive nitrogen species (RNS), particularly nitrite and nitrate, compared to argon-only discharges. Reactive species concentrations obtained using test strips (Table 3) reflect ranges from three independent PAL preparations. Due to the semiquantitative nature of the colorimetric detection method and the use of manufacturer-defined concentration intervals, the results were evaluated descriptively without formal statistical comparison between groups.

**Table 3:** Concentrations (mg/L) of Reactive Oxygen and Nitrogen Species in the Plasma-Activated Liquids^a^

groups	ozone (mg/L)	H_2_O_2_ (mg/L)	nitrite (mg/L)	nitrate (mg/L)
D1	0.03	2.0	25–50	1–5
D2	0.86	0.5	250	40
D3	1.22	0.5	250	40–80
S1	0.02	2.0–5.0	10–25	1–5
S2	0.76	0.5	250–500	80
S3	1.76	0.5	250–500	80

a Legend: H_2_O_2_: hydrogen peroxide; NO_2_^–^: nitrite; NO_3_^–^: nitrate; O_3_: ozone; D1: distilled water activated by argon plasma; D2: distilled water activated by argon and compressed air plasma; D3: distilled water activated by compressed air plasma; S1: Saline solution activated by argon plasma; S2: Saline solution activated by argon and compressed air plasma; S3: Saline solution activated by compressed air plasma.

### 3.4 Antifungal Activity against *C. albicans* Planktonic Cells

Figure 5 presents the antifungal activity of PALs, generated using both distilled water and saline solution under different plasma gas compositions (argon, argon + air, and air) and flow rates (1.5 or 3.0 L/min), against *C. albicans* planktonic cells. The PALs were applied under varying exposure times (10 or 30 min), including a condition with aged PALs stored for 24 h.

**Figure 5:**
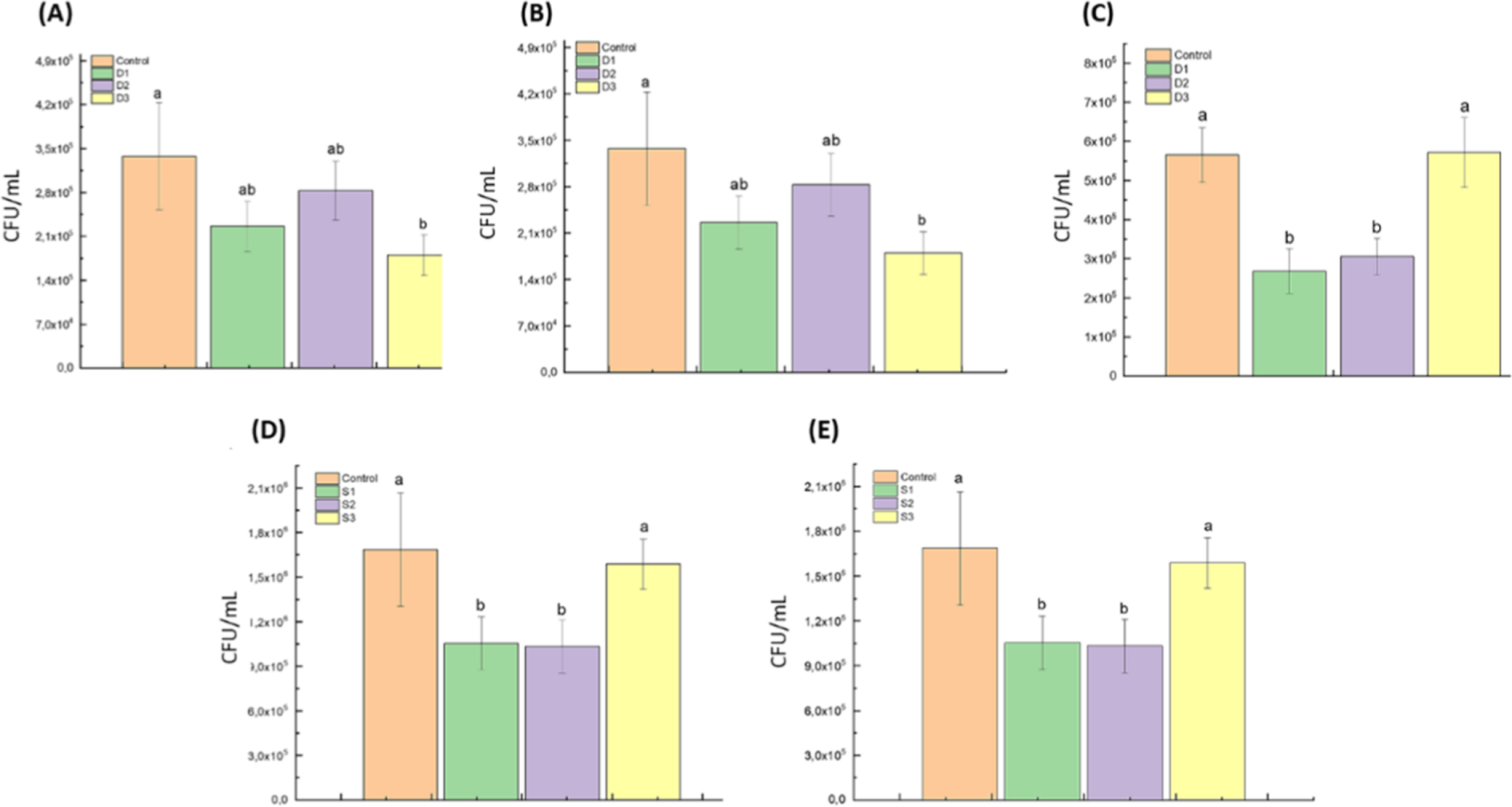
Antifungal activity of plasma-activated liquids (PALs) against *C. albicans* planktonic cells. Colony-forming unit (CFU/mL) values are shown after different exposure conditions. Bars with different letters indicate statistically significant differences (*p* < 0.05). (a) Distilled water PALs generated at 1.5 L/min and applied for 30 min; (b) distilled water PALs generated at 3.0 L/min and applied for 10 min; (c) aged distilled water PALs (24 h) generated at 3.0 L/min and applied for 30 min; (d) Saline PALs generated at 3.0 L/min and applied for 10 min; (e) Saline PALs generated at 3.0 L/min and applied for 30 min. Group labels: D1: distilled water activated by argon plasma; D2: by argon + air plasma; D3: by air plasma; S1: saline activated by argon plasma; S2: by argon + air plasma; S3: by air plasma. Statistical analysis was performed using One-way ANOVA followed by Tukey’s post hoc test.

When distilled water was used and a gas flow of 1.5 L/min was applied for 30 min (Figure 5a), all experimental groups demonstrated reduced CFU/mL values. The most pronounced reduction was observed in group D3 (compressed air plasma), with a 46.66% decrease (*p* < 0.05), followed by D1 (argon plasma, 33.12%) and D2 (argon + air, 16.19%), although only D3 reached statistical significance. No significant differences were detected among D1, D2, and D3. In contrast, when the gas flow was increased to 3.0 L/min and the exposure time reduced to 10 min (Figure 5b), none of the distilled water groups (D1, D2, or D3) showed significant reductions in CFU/mL (*p* > 0.05), indicating a reduced antimicrobial effect under these conditions. Notably, PALs generated at 3.0 L/min and stored for 24 h (Figure 5c) retained substantial antifungal activity, with D1 and D2 showing significant reductions of 53% and 46%, respectively (*p* < 0.05), while D3 showed no significant effect. These results suggest that PALs produced with argon or argon–air mixtures may retain biologically active RONS over time, contributing to prolonged antimicrobial effects. When saline solution was used as the base liquid and the PALs were applied for 10 min (Figure 5d), both S1 (argon plasma) and S2 (argon + air plasma) resulted in significant reductions in CFU/mL (37.45% and 38.6%, respectively; *p* < 0.05), with no difference between them. However, S3 (compressed air plasma) exhibited only a 9.95% reduction, which was significantly less effective than S1 and S2. Under prolonged exposure of 30 min using saline PALs (Figure 5e), only S1 achieved a statistically significant reduction (52%, *p* < 0.05). Although S2 resulted in a 46% decrease, it did not reach statistical significance, and S3 remained ineffective compared to the control. Collectively, these results indicate that both the liquid matrix and plasma gas composition critically influence the antifungal performance of PALs. Argon and argon–air plasmas applied to either distilled water or saline solution consistently demonstrated superior antimicrobial activity, while compressed air alone (D3/S3) showed limited efficacy, particularly in the saline environment.

### 3.5 Inhibitory Effects on *C. albicans* Biofilms

Figure 6 shows the inhibitory effects of PALs, generated using distilled water and 0.9% saline solution with argon plasma (3.0 L/min), on *C. albicans* biofilms after 24 and 48 h of contact.

**Figure 6:**
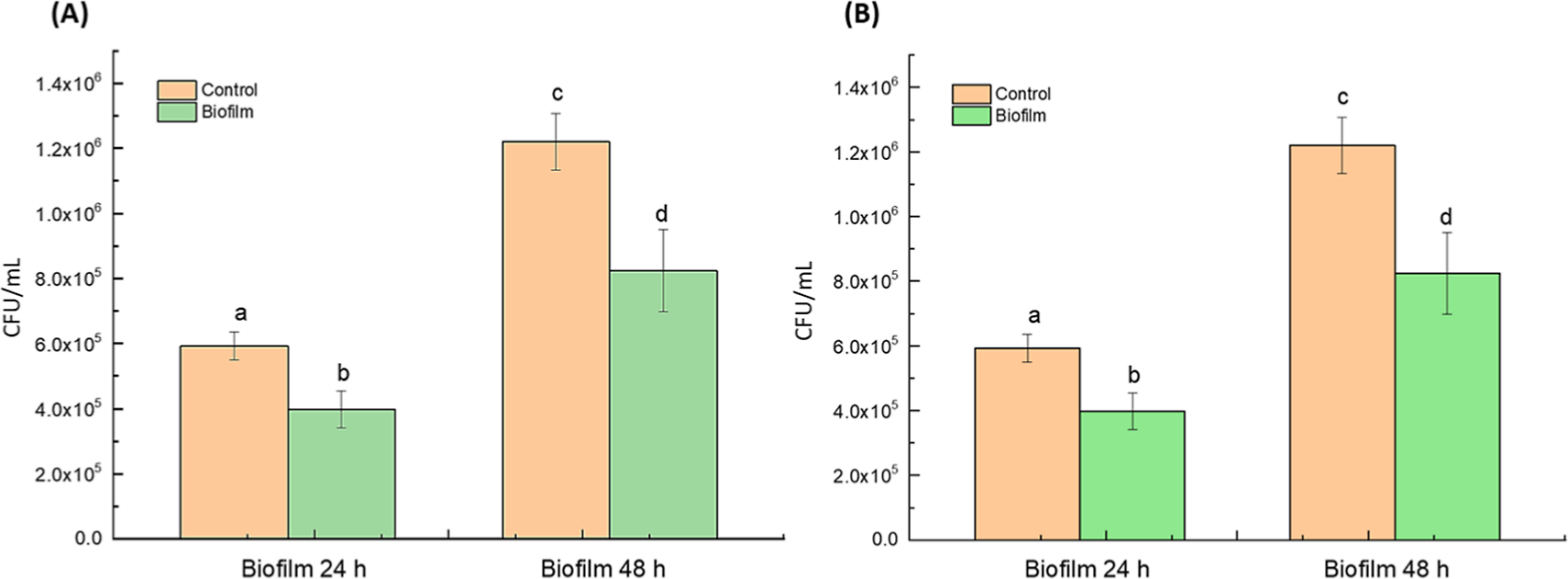
Inhibitory effect of plasma-activated liquids (PALs) on *C. albicans* biofilms. Viable cell counts (CFU/mL) after 30 min exposure to PALs generated with argon plasma (3.0 L/min) in (a) distilled water (D1 group); (b) 0.9% saline solution (S1 group). Bars represent means ± standard deviation. Different letters indicate statistically significant differences (*p* < 0.05) among groups. Statistical analysis was performed using One-way ANOVA followed by Tukey’s post hoc test.

In both cases, the PALs were applied for continuous exposure, and viable cell counts were assessed at each time point. As shown in Figure 6a, distilled water PALs (D1 group) significantly reduced CFU/mL after both 24 h (32.86%) and 48 h (32.37%) exposures compared to the control (*p* < 0.05). Similarly, in Figure 6b, saline-based PALs (S1 group) resulted in a 45.10% reduction in 24 h and a 32.45% reduction at 48 h, with both effects being statistically significant (*p* < 0.05). These findings demonstrate that argon-activated PALs maintain antifungal efficacy over prolonged contact times and are effective in reducing biofilm associated with *C. albicans* cells in both distilled water and saline solution. The slightly greater reduction observed with saline PALs at 24 h suggests a possible enhanced short-term activity, while both types maintained comparable effects at 48 h. These results reinforce the potential of PALs as stable, long-acting agents against fungal biofilms.

### 3.6 Cytotoxicity of Plasma-Activated Liquids

As shown in Figure 7, all PALs demonstrated low cytotoxicity. Vero cell viability remained above 70% immediately after exposure and after 24 h, for both distilled water and saline PALs. These results comply with the ISO 10993-5:2009 threshold for noncytotoxicity.

**Figure 7:**
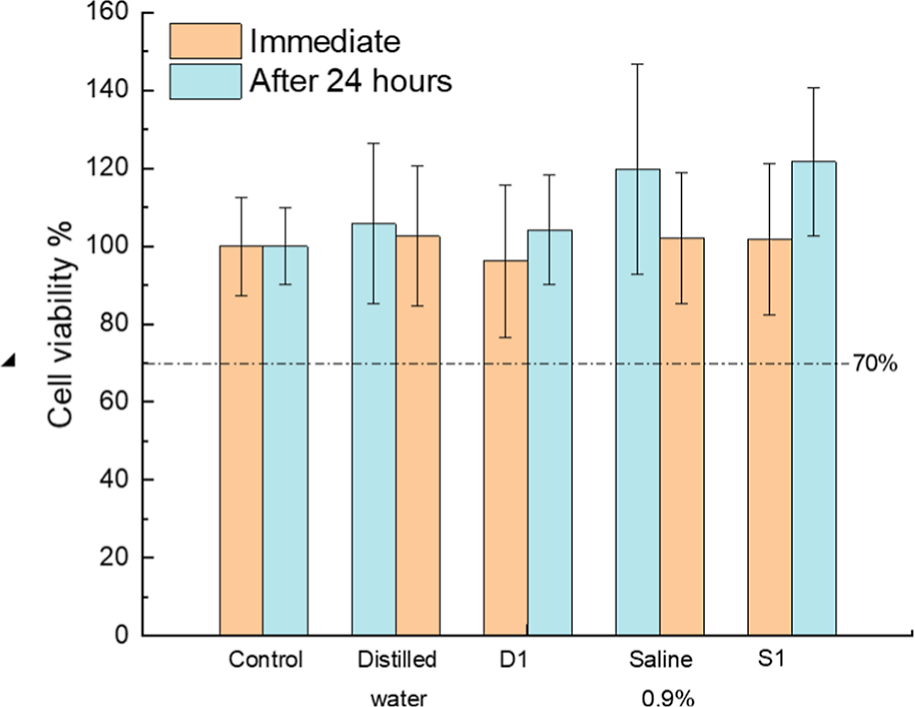
Evaluation of cytotoxicity of plasma-activated liquids (PALs) on Vero cells. Cell viability (%) measured immediately and 24 h after 30 min exposure to nonactivated and plasma-activated liquids. D1: distilled water activated with argon plasma; S1:0.9% saline solution activated with argon plasma. The dashed line at 70% indicates the threshold for cytotoxicity according to ISO 10993-5:2009. All values remained above the cytotoxicity limit, indicating acceptable biocompatibility. Statistical analysis was performed using One-way ANOVA followed by Tukey’s post hoc test.

## 4 Discussion

Several studies have demonstrated that different variables affect the composition of PALs, such as the activation duration, gas flow rate, and gas composition. From the evaluation of the physicochemical parameters measured, it was observed that using a lower gas flow rate (1.5 L/min) led to changes in the physicochemical properties of the liquids, albeit not as pronounced as those obtained with activation at 3.0 L/min. These changes, summarized in Table 2 and Figure 2, indicate the formation of reactive oxygen and nitrogen species (RONS).

Electrical conductivity analysis is a parameter used to check the concentration of reactive ions in water.^42,86–88^ In our findings, it was observed that after the activation of distilled water by plasmas of different gases, an increase in conductivity was recorded.

Increasing the gas flow rate to 3.0 L/min (Table 2, Figure 2b) caused a more substantial increase in conductivity, which was accompanied by the presence of long-lived RONS such as hydrogen peroxide (H_2_O_2_), nitrate (NO_3_^–^), nitrite (NO_2_^–^), and ozone (O_3_), as demonstrated by UV–vis deconvolution analysis (Figures 3 and 4) and RONS quantification (Table 3). These results corroborate the findings of Lamichhane et al.^89^ and Xia et al.,^88^ who observed a proportional increase in electrical conductivity with RONS concentration following argon plasma activation.

Groups D2, D3, S2, and S3, which exhibited the highest conductivity values (Table 2), also showed the highest concentrations of nitrate, nitrite, and ozone (Table 3), highlighting the influence of gas composition and flow rate on the chemical profile of PALs.

Another parameter linked to RONS formation is the oxidation–reduction potential (ORP), which reflects the solution’s ability to oxidize or reduce other substances and serves as a rapid indicator of disinfection potential.^87^ An increase in ORP was observed in all PAL groups at both 1.5 and 3.0 L/min (Table 2, Figure 2), consistent with previous reports.^88,90^ According to Thirumdas et al.^87^ and Ma et al.,^77^ higher ORP values correspond to greater oxidizing capacity and enhanced antimicrobial potential.

Some authors^20,28,91,92^ have reported that high ORP and low pH are indicative of elevated RONS concentrations in PALs. Acidic conditions favor the formation and stability of certain species.^76,79,80,93,94^ In our results, groups D2, D3, S2, and S3 showed pH reductions of 49.0%, 51.3%, 45.8%, and 47.8%, respectively, and also the highest ORP increases (Table 2), with increments of 70.4% (D2), 76.6% (D3), 51.1% (S2), and 52.1% (S3).

TDS also increased postactivation in all groups (Table 2), suggesting the formation of new chemical species. Similar behavior was described by Miranda et al.^81^ and Sampaio et al.^20^ TDS served as another proxy for RONS concentration, with D2, D3, S2, and S3 showing the highest values compared to the control and D1/S1 groups (Figure 1).

Physicochemical parameters (Table 2, Figure 2) effectively screen for RONS, as higher conductivity, TDS, and ORP with lower pH signal increased concentrations. This inverse relationship was clear in groups D1 and S1, which showed lower RONS levels but higher biological efficacy (Table 3).

Regarding antifungal activity, studies evaluating PALs against *C. albicans* are limited, and those that exist report minimal fungal inactivation.^28,81,95^ The lower sensitivity of fungi compared to bacteria may be attributed to their complex cell wall structure and intracellular antioxidant enzymes such as superoxide dismutase (SOD) and catalase, which help mitigate oxidative stress.^66,96,97^

Among the few reports, Ercan et al.^78^ and Laurita et al.^80^ demonstrated antifungal effects against *C. albicans*, but using direct plasma exposure in fungal suspensions, which differs from the indirect PAL approach used in our study. These models likely include short-lived species critical for initial microbial reduction.

Our results demonstrate that specific PALs, particularly those generated with argon plasma (D1 and S1), exhibited significant antifungal activity against *C. albicans* (Figure 5). However, current exposure times (10 and 30 min) may be limiting for clinical applications. The aged PALs (24 h) also retained activity (Figure 5c), supporting the potential for preprepared formulations. Future work should focus on optimizing activation and exposure parameters or developing slow-release delivery systems to enable prolonged antifungal activity.

At 1.5 L/min, only the D3 group showed a statistically significant reduction in *C. albicans* (Figure 5a), which, although similar to D1 and D2 in RONS content (Table 3), exhibited inconsistent antifungal results. This highlights that while RONS were formed, their concentration or reactivity was insufficient to achieve consistent inactivation.

Increasing the flow rate to 3.0 L/min significantly enhanced antifungal activity (Figure 5c–e), indicating that both flow rate and gas composition influence the generation and efficacy of RONS (Table 2; Figures 3 and 4).

Groups treated with argon plasma (S1, D1, S2, D2) demonstrated the best antifungal effects (Figure 5c,e), likely due to higher H_2_O_2_ concentrations (Table 3). Vlad et al.^33^ reported similar findings, where argon-generated PALs exhibited higher peroxide levels than helium or air plasmas. However, D1 and D2 were only effective at 30 min, suggesting that extended contact is required for antifungal efficacy (Figure 5c).

Xia et al.^88^ also showed that argon plasma results in higher H_2_O_2_ production than compressed air. In our spectrophotometric analysis (Figures 3 and 4), H_2_O_2_ bands were not prominent in S2 and S3, confirming the qualitative nature of the method but also its usefulness for initial screening.

Different studies have demonstrated that hydrogen peroxide is one of the main contributors to the antimicrobial activity of plasma-treated liquids,^33,76,98,99^ primarily due to its capacity to react with nitrite and nitrate to generate peroxynitrite.^33,80,99^ Additionally, peroxynitrite can be produced from the reaction between hydrogen peroxide and nitric acid.^76,80^ Peroxynitrite has been identified as one of the key antimicrobial byproducts generated in LAPs, even at low concentrations.^33,79,80,98^ Its antimicrobial efficacy is associated with its ability to oxidize, nitrate, and hydroxylate biomolecules under physiological conditions, thereby exerting cytotoxic effects.^79,100^

Moreover, peroxynitrite can readily penetrate biological membranes, as its permeability coefficient is comparable to that of hydrogen peroxide, enabling it to act as an effective intracellular oxidant. Upon entry, ONOO^–^ can induce lipid and protein peroxidation and nitration, either directly or through its decomposition into reactive species such as •OH and NO_2_. These radicals contribute to oxidative stress by compromising membrane integrity, forming transient pores, and facilitating further RONS penetration and intracellular accumulation.^79,100^

While our findings demonstrate a correlation between the antifungal activity of specific PAL groups and their elevated concentrations of hydrogen peroxide and inferred peroxynitrite, the underlying cellular mechanisms remain to be conclusively established. In this study, peroxynitrite formation was inferred based on chemical signatures and literature-supported reaction pathways, but no direct mechanistic assays were conducted to confirm its role in fungal inactivation. As such, the observed associations between RONS content and antifungal efficacy should be interpreted as correlative rather than causative.

Future investigations should incorporate targeted mechanistic assays, including ROS scavenger experiments (e.g., catalase or N-acetylcysteine), membrane integrity assessments (e.g., propidium iodide staining, LDH release), and transcriptomic or proteomic profiling of *C. albicans* exposed to PALs. These complementary approaches will be essential to elucidate the specific pathways and molecular targets involved in PAL-induced fungal cell death and to substantiate the contributions of key reactive species such as H_2_O_2_ and ONOO^–^.

According to Bauer,^21^ Liu et al.,^76^ and Zhou et al.,^79^ increased concentrations of hydrogen peroxide correlate with higher levels of peroxynitrite and hydroxyl radicals. These secondary radicals originate from hydrogen peroxide decomposition and promote additional peroxynitrite formation. Thus, it is likely that the combination of hydrogen peroxide and peroxynitrite contributed to the enhanced antifungal performance observed in groups S1 and D1 (Figure 5c,e).

Xia et al.^88^ reported elevated nitrite and nitrate levels in water activated using compressed air plasma. Similar trends were observed in this study for groups D2, S2, D3, and S3 (Table 3), likely due to the nitrogen and oxygen content of compressed air.

Despite these physicochemical modifications, LAPs generated solely from compressed air plasma (S3 and D3) showed no significant antifungal activity against *C. albicans* (Figure 5a,d,e), even though they presented increased levels of nitrite and nitrate (Table 3).

Ozone (O_3_) was another reactive species detected in higher concentrations in groups D3 and S3 (Table 3). These findings are consistent with Laurita et al.,^80^ who reported that increased nitrate levels are typically accompanied by reduced nitrite and hydrogen peroxide concentrations. Moreover, nitrite can react with dissolved ozone and hydrogen peroxide to form nitrate, which is more stable and less reactive than nitrite.^88^ While nitrite and nitrate are considered weakly reactive, they act as precursors to more potent intermediates such as peroxynitrite, especially under acidic conditions.^77,93^

The results of this study underscore the complex chemistry underlying LAPs. High concentrations of specific species do not necessarily translate into effective antimicrobial activity, as evidenced in D2, S2, and S3 (30 min contact; Figure 5e), and D3 and S3 (10 min contact; Figure 5a,d). Interactions between components can yield secondary products lacking antimicrobial potency. Ma et al.^77^ showed that nitrite, nitrate, and hydrogen peroxide in acidic environments failed to exert bactericidal activity against *E. coli* unless synergistically combined with low pH and plasma-generated intermediates like nitrogen peroxide.

Although some LAP groups did not exhibit effective antifungal activity against *C. albicans*, a noteworthy study by Liu et al.^101^ demonstrated that plasma-treated liquids increased the permeability of *Saccharomyces cerevisiae* cells, thereby enhancing the efficacy of sodium lauryl sulfate, which alone had no antifungal effect. These results suggest that LAPs may sensitize fungal cells to conventional agents, reducing drug concentration requirements and potential toxicity.

This study also demonstrated that LAPs retained antifungal activity after being frozen for 24 h (Figure 5c), in agreement with Figueira et al.,^102^ Traylor et al.,^98^ and Vlad et al.,^33^ who reported preserved antibacterial activity for LAPs stored for up to 7 days. According to Thirumdas et al.^87^ and Tsoukou et al.,^94^ antimicrobial activity has been retained even after 30 days at −80 °C.

Figueira et al.^102^ also noted that storage at low temperatures (∼3 °C) better preserved physicochemical parameters such as pH and conductivity, which are closely linked to the stability of reactive species, as confirmed by the present findings (Table 2).

After confirming antifungal action on planktonic *C. albicans* cells, we evaluated LAP efficacy against fungal biofilms. Given the promising results obtained for D1, D2, and S1 (Figure 5c,e), only argon-based LAPs were selected for biofilm assays.

LAPs derived from distilled water activated with argon plasma effectively reduced the viability of both 24 h and mature (48 h) biofilms (Figure 6a). In contrast, LAPs from saline activated with argon were only effective against mature biofilms, not the 24 h form (Figure 6b).

Considering possible in vivo applications, cytotoxicity assays on Vero cells indicated that argon-based LAPs (D1 and S1) maintained cell viability above 70% both immediately and after 24 h (Figure 7), confirming their nontoxic profile. Nonetheless, the present study did not include direct head-to-head comparisons with standard antifungal drugs under identical conditions, which limits the clinical contextualization of LAP efficacy. In future experiments, we plan to include standard-of-care agents, such as fluconazole, amphotericin B, nystatin, and clotrimazole, tested in parallel with LAPs on both planktonic and biofilm forms, following CLSI protocols for MIC/MFC and MBEC determination. These assays will allow us to benchmark LAP performance against established therapies, explore potential synergistic or additive effects, and better define clinically relevant dosing strategies.

Overall, the results demonstrated that LAPs are effective against *C. albicans* in both planktonic and biofilm states following 10- and 30 min exposures (Figures 5 and 6). To our knowledge, this is the first report to describe LAP effects on *C. albicans* biofilms. However, these treatment durations may limit clinical applicability, particularly in oral settings where prolonged continuous exposure is not practical. In our assays, optimized PALs (e.g., S1 and S2 at 3.0 L/min) already achieved ∼37–39% CFU reduction within 10 min, suggesting that further gains are possible through delivery optimization. Strategies to reduce clinical application time include: (i) using mucoadhesive hydrogels or thin polymeric films (e.g., chitosan/Carbopol, thermos responsive poloxamers) preloaded with PAL to maintain local release over 30–60 min while requiring only brief placement; (ii) spray or nebulized delivery to reach difficult intraoral niches with short bursts; (iii) presoaked mucoadhesive swabs, gauze, or custom oral trays/splints acting as reservoirs to extend contact without continuous handling; and (iv) combining PAL with standard antifungals to exploit potential synergistic effects and reduce required contact time. The demonstrated stability of PALs after 24 h frozen storage supports prepreparation of unit doses for such delivery systems. Future work should focus on optimizing exposure times, evaluating these prolonged-contact delivery platforms in vitro and in vivo, or using LAPs as carriers for antifungal agents.

## 5 Conclusion

This study demonstrates the antifungal potential of PALs generated via gliding arc discharges using argon and air-based gas compositions in distilled water and saline matrices. Among the experimental groups, PALs activated with argon (D1 and S1) consistently exhibited selective antifungal activity against *C. albicans* in both planktonic and biofilm forms, while maintaining mammalian cell viability above cytotoxicity thresholds. These biological effects were associated with elevated concentrations of hydrogen peroxide and the inferred formation of secondary reactive species, particularly peroxynitrite, which are proposed to contribute to membrane disruption and oxidative stress.

The observed physicochemical alterations, including increased conductivity, ORP, and TDS, alongside reduced pH, correlated with the presence of long-lived RONS. However, antifungal efficacy was not determined solely by overall RONS concentration: despite higher levels of nitrite and nitrate, PALs generated with air plasma (D3 and S3) demonstrated limited antifungal effects, emphasizing that the chemical identity and interplay of reactive species critically influence biological outcomes. Notably, PALs retained antifungal efficacy after 24 h of frozen storage, underscoring their physicochemical stability and clinical handling potential.

Importantly, this is the first study to systematically evaluate the effects of PALs on *C. albicans* biofilms, a clinically relevant and treatment-resistant fungal phenotype. The ability of argon-based PALs to significantly reduce biofilm viability, while preserving host cell compatibility, supports their development as adjunctive or alternative antifungal therapies, especially for oral candidiasis in immunocompromised populations.

Future research should aim to (i) optimize plasma activation parameters to reduce required exposure times; (ii) investigate synergistic combinations with existing antifungal agents; (iii) explore advanced delivery systems such as mucoadhesive films or hydrogel carriers for prolonged mucosal contact; and (iv) incorporate mechanistic assays, including ROS scavenger experiments, membrane integrity assessments, and transcriptomic or proteomic analyses, to validate the causal role of specific RONS in fungal inactivation. Collectively, these findings position PALs as a promising and versatile platform for nonconventional antifungal therapies targeting drug-resistant and biofilm-associated infections.
